# Late Replication of the Inactive X Chromosome Is Independent of the Compactness of Chromosome Territory in Human Pluripotent Stem Cells

**Published:** 2013

**Authors:** A. V. Panova, E. D. Nekrasov, M. A. Lagarkova, S. L. Kiselev, A. N. Bogomazova

**Affiliations:** Vavilov Institute of General Genetics, Russian Academy of Sciences, Gubkina Str., 3, Moscow, Russia, 119991

**Keywords:** reprogramming, ESCs, iPS cells, chromosome territories, the X chromosome, late replication

## Abstract

Dosage compensation of the X chromosomes in mammals is performed via the formation of facultative heterochromatin on extra X chromosomes in female somatic cells. Facultative heterochromatin of the inactivated X (Xi), as well as constitutive heterochromatin, replicates late during the S-phase. It is generally accepted that Xi is always more compact in the interphase nucleus. The dense chromosomal folding has been proposed to define the late replication of Xi. In contrast to mouse pluripotent stem cells (PSCs), the status of X chromosome inactivation in human PSCs may vary significantly. Fluorescence *in situ* hybridization with a whole X-chromosome- specific DNA probe revealed that late-replicating Xi may occupy either compact or dispersed territory in human PSCs. Thus, the late replication of the Xi does not depend on the compactness of chromosome territory in human PSCs. However, the Xi reactivation and the synchronization in the replication timing of X chromosomes upon reprogramming are necessarily accompanied by the expansion of X chromosome territory.

## INTRODUCTION

The chromatin structure and architecture of the nucleus are the crucial elements in the regulation of transcription and replication, the key genetic processes that occur in nuclei. Сhromosomes occupy certain non-overlapping regions in the interphase nucleus, forming the so-called chromosome territories. The densely packed chromatin mostly localizes in the peripheral and perinucleolar regions of the nucleus [1]. The replication of dispersed euchromatin and densely packed heterochromatin is separated both in space and in time. Dispersed euchromatin replicates during the early S-phase, while the condensed heterochromatin replicates in the late S-phase [2]. T. Ryba *et al*. have put forward a hypothesis that late replication of densely packed chromatin domains can be attributed to the fact that access for the replication initiation factors to these regions is hindered [3].

An inactivated X chromosome (Xi), which becomes transcriptionally silent as a result of the dosage compensation, forms the compact structure known as the Barr body on the nuclear periphery and replicates in the late S-phase, is an example of a bulk heterochromatin domain inside the nucleus in female mammalian somatic cells [4].

The variability of the status of X chromosome inactivation in female human pluripotent stem cells (PSCs) provides an interesting opportunity for studying the relationship between the different epigenetic states of chromatin, the architecture of the chromosome territories in the interphase nucleus, and the regulation of replication [5–7].

Up to now, female human embryonic stem cell (hESCs) lines with two active X chromosomes (Xa), one inactivated X chromosome, and hESC lines without any conventional cytological indicators of X inactivation have been described. As for human induced pluripotent stem cells (iPSCs), there is no clear opinion about the possibility of complete reactivation of the X chromosome and the possibility of long-term maintenance of the active status *in vitro*. However, the X chromosome during reprogramming undoubtedly undergoes a number of significant epigenetic changes associated at least with partial reactivation [8–10]. Our study was aimed at searching for a relationship between the replication timing of the X chromosomes in human PSCs with different statuses of X chromosome inactivation and the degree of compactness of their chromosome territories.

Our results demonstrate that replication of the inactive X chromosome in the late S-phase of the cell cycle can be unrelated to the compactness of the chromosome area and that the late-replicating and transcriptionally silent Xi can be present in female PSCs in the relaxed state. Nevertheless, the X chromosome territory relaxes as the X chromosome becomes active, which is accompanied by synchronization of the replication of homologous X chromosomes.

## MATERIALS AND METHODS

**Cell cultures**

Human ESC lines hESM01 and hESM04 have been described earlier [11]. The cell line HUE S 9 was created and kindly provided by D. Melton (Harvard University, USA) [12]. A human umbilical vein endothelial cell (HUVEC ) line was obtained in accordance with [13]. iPSC lines (incompletely reprogrammed clones iPS-6 and iPS-7 and completely reprogrammed clone iPS-12) were obtained from HUVEC cells by lentiviral transfection with four transcription factors (KLF4, OCT 4, SOX2 and C-MYC) [14] and described in [15]. The iPSC MA-02 line was obtained from dermal fibroblasts according to the previously described procedure [16].

PSC lines were cultured in mTeSR1 medium (Stem- Cell Technologies) on Petri dishes coated with BD Matrigel. The HUVEC line was cultured in DMEM/F12 supplemented with 15% FBS, 5 ng/ml hrbFGF (Peprotech), 20 ng/ml hrVEGF (Peprotech), 1% nonessential amino acids, 2 mM *L*-glutamine, 50 U/ml penicillin, and 50 ng/ml streptomycin (all reagents purchased from Hyclone). All cell lines were cultured in 5% CO_2_ at 37°C.

**Immunostaining**

The nuclei and metaphase chromosomes were immunostained as described in [8]. The following primary antibodies were used: polyclonal rabbit anti-H3K27me3 antibodies (Millipore, dilution 1 : 500); polyclonal rabbit anti-H3K4me2 antibodies (Abcam, 1 : 200); monoclonal mouse anti-H3K4me2 antibodies (Abcam, 1 : 100); and polyclonal rabbit anti-H3K9me3 antibodies (Abcam, 1 : 200). Alexa Fluor 546 secondary goat anti-rabbit IgG antibodies (Invitrogen, 1 : 1000) or Alexa Fluor 488 goat anti-mouse IgG antibodies (Invitrogen, 1 : 1000) were also used. The DNA of nuclei and metaphase chromosomes was stained with DAPI; Vectashield solution (Vector Laboratories) was then spotted onto the specimen, and the specimen was covered with a cover slip.

## RNA FISH

RN A FISH was conducted using fluorescently labeled DNA probes derived from BAC-clones (Empire Genomics) according to the procedure described in [17]. The following BAC clones were used: RP11-13M9 for the *XIST *locus and RP11-1104L9 for the *POLA1 *locus.

***In situ *hybridization with whole chromosome probe (painting)**

In order to prepare the specimens of interphase nuclei, cells were treated with trypsin (0.05% Trypsin, Hyclone). Trypsin was inactivated using FBS (Hyclone), and cells were treated with a hypotonic solution (0.075 M KCl) for 18 min at 37°C. The cells were fixed using two fixatives (6 : 1 and 3 : 1 mixtures of methanol and glacial acetic acid, respectively). The fixed cells were stored in the 3 : 1 fixative at –20°C. The suspension of fixed cells was dropped onto ice-cold wet glass slides and air-dried for 24 h. In order to improve FISH quality, the specimens were incubated in 0.25% paraformaldehyde for 10 min at room temperature; the pretreated specimens were sequentially dehydrated in 70, 80, and 96% ethanol, followed by treatment with 0.002% pepsin solution (Sigma) in 0.01 M HCl for 30 s at 37°C and another cycle of dehydration in ethanol. Denaturation was carried out in 70% formamide in the 2xSSC buffer for 5 min at 75°C, followed by dehydration in ethanol. Whole chromosome probes to chromosomes X and 8 were purchased from MetaSystems. The specimens were subsequently stained with DAPI; the Vectashield solution (Vector Laboratories) was then spotted onto the specimen, and the specimen was covered with a cover slip.

**Detection of the replication timing using 5-bromo-2-deoxyuridine (BrdU)**

The cells were incubated in the presence of 5-bromo- 2-deoxyuridine at a final concentration of 10 μM for 20 min 6–10 h prior to fixation. Colcemid (Demicolcine solution, Sigma) at a final concentration of 0.2 μg/ml was added to the cultivation medium 1 h prior to fixation. The metaphase spreads were prepared according to the above-described procedure for interphase nuclei. For DNA denaturation, the metaphase spreads were treated with 70% formamide in the 2x SSC buffer for 5 min at 75°C. Next, they were dehydrated in ethanol and incubated with primary mouse anti-BrdU antibodies (Sigma, 1 : 1000) for 2 h at 37°C. The cells were washed in a PBS-0.1% Tween 20 solution. The specimens were subsequently incubated with Alexa Fluor 546 sec ondary goat anti-mouse IgG antibodies (Invitrogen, 1 : 1000) for 1 h at room temperature and stained with DAPI. The X chromosome was identified on the basis of inverted DAPI-banding or X-specific FISH probe.

**Microscopy and photography**

The metaphase spreads and interphase nuclei were analyzed on a Axio Imager A1 epifluorescence microscope (Carl Zeiss). Pseudo color images of the microobjects were obtained using the AxioVision software (Carl Zeiss).

**Comparison of the degrees of chromosome compactness, calculation of variance**

In order to objectively compare the compactness of two X chromosomes in each individual cell we used software that had been designed especially for this experiment. The calculation algorithm is explained below.

Let us consider the channel of a micro-image of interphase nuclei, where the data on the fluorescence intensity of the stained chromosome territories is stored.

Let us assume that *x*, *y *are the coordinates on the plane formed by the points of the image, and *P_s_*(*x*, *y*) is the intensity of an image point as a function of its coordinate. First, the background level of fluorescence was intercepted by a linear transformation:

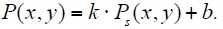



Let us denote the image area where the chromosome territory under analysis lies as G. The parameters *P*_0_,* x*_0_, *y*_0_ were calculated for each chromosome territory as follows:

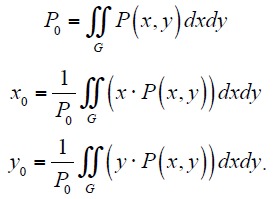



Next, substitution of variables *P*(*x*, *y*) → *P*(*r*, φ) was performed according to the transformation formulas given below:

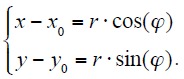



This substitution of variables is nothing but a transition to polar coordinates with the center at point (*x*_0_,* y*_0_).

Function *P*(*r*, φ) was then averaged over the variable φ so that *P*(*r*, φ) → *P*(*r*). The formula used for averaging is presented below.

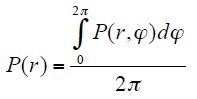



Function *P*(*r*) was normalized by *P*(*r*) → *P_n_*(*r*).

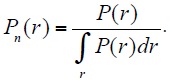



Function *P_n_*(*r*) was approximated to the normal distribution of *N*(*r*) using the least-squares procedure:

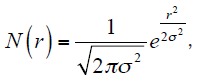



in other words, a particular σ value was selected, for which

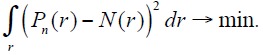



The parameter σ^2^ is the variance of the normal distribution. This very parameter was output by the software as the analysis result and was subsequently used to estimate the compactness of chromosome territories. The σ^2^ values for each X chromosome image in each nucleus in all cell lines were obtained in this study.

The ratio between a chromosome territory with a higher σ^2^ value and a chromosome territory with a lower σ^2^ value was individually calculated for each nucleus. The comparison of the σ_1_^2^ / σ_1_^2^ values for two autosomes in the same cell was used as a control and to determine the threshold values of the ratio between the variance of two chromosomes σ_1_^2^ / σ_1_^2^ . The threshold value σ_1_^2^ / σ_1_^2^ = 2.1 was obtained by comparing autosomes. Thus, all cells with the σ_1_^2^ / σ_1_^2^ value ≤ 2.1 for the X chromosomes were considered to possess two dispersed chromosome territories. An analysis of the ratio between X chromosome dispersions in pluripotent cells (the line that has previously been described as a line with one inactive X chromosome, hESM04 [11]) allowed to reveal the second threshold value σ_1_^2^ / σ_1_^2^ = 3. Thus, all the cells with a σ_1_^2^ / σ_1_^2^ value ≥ 3 for the X chromosomes were considered to possess one compact and one dispersed chromosome territory. The data obtained by this analysis are listed in *Table 2*. A total of 50–100 nuclei were analyzed in each cell line.

**Table 1:** Summary of X chromosome inactivation in the human pluripotent and somatic cell lines used.

Cell line	H3K4me2 Euchromatin mark	H3K27me3 Heterochromatin mark	H3K9me3 Heterochromatin mark	RN A-FISH		X inactivation status
				XIST-coating	Expression of POLA1	
HUES9	+	-	Some bands were immunopositive	-	Biallelic	XаXa*
hESM01	+	-	+	-	Monoallelic	XaXi *
hESM04	-	+	+	+	Monoallelic	XaXi
iPS MA02	+	-	-	-	Biallelic	XаXa
HUVEC	-	+	+	+	Monoallelic	XaXi
IPS-6	-	+	+	+	Monoallelic	XaXi
IPS-7	-	+	+	+	Monoallelic	XaXi
IPS-12	+	+	+	+	Monoallelic	XaXi

* XaXa – two active X chromosomes in cells; XaXi – one active and one inactivated X chromosome in cells.

## RESULTS AND DISCUSSION

**Characterization of the status of X chromosome inactivation in human ESCs and iPSCs**

The status of the Х chromosome in all the cell lines used in this study was characterized previously using the regular criteria: presence/absence of the XIST-RN A cloud in the interphase nucleus, presence/absence of focal staining with anti-H3K27me3 antibodies in the interphase nuclei; and monoallelic/biallelic expression of the *POLA1 *gene. The data, including those published earlier for some cell lines [8, 11], are summarized in *Table 1*. Monoallelic or biallelic expression of the *POLA1* gene was the main and determining criterion of the X chromosome status.

It is clear from *Table 1* that identically to the original somatic cells, the incompletely reprogrammed (the so-called imperfect iPSC clones, iPS-6 and iPS-7) cells exhibited all the features of the Xi: the XIST-RN A cloud and focus of H3K27me3 in the interphase nucleus; in addition, monoallelic expression of the *POLA1* gene was observed in these cells. The completely reprogrammed clone iPS-12 exhibited the features of partial reactivation (the presence of H3K4me2 on both X chromosomes) [8] but was characterized by monoallelic* POLA1 *expression. The ESC line hESM01 contained the transcriptionally silent X; however, it had lost such inactivation markers as the XIST-RN A cloud and focus of H3K27me3 in the interphase nuclei.

Based on all these criteria, the iPSC line MA-02 and ESC line HUE S 9 can be classified as lines with Xa. Neither the XIST cloud was detected via RN A-FISH in all these cell lines nor focal staining with anti-H3K27me3 antibodies in the interphase nuclei. Staining with antibodies against the active chromatin marker H3K4me2 on both X chromosomes and biallelic expression of the* POLA1 *gene were observed. It should be mentioned that X chromosome reactivation during the reprogramming of human cells is a rather infrequent event. Most clones produced by regular reprogramming have a single Xi [9, 18, and our own observations].

An analysis of the replication timing of the X chromosomes was carried out for all the ESC and iPSC cell lines listed in *Table 1*.

**Replication timing of the X chromosomes**

Late replication is known to be typical of heterochromatin; namely, replication in the late S-phase of the cell cycle after the euchromatin replication. In particular, it is also typical of facultative heterochromatin of the Xi [2]. In order to determine the replicating timing of the X chromosomes in the S-phase of the cell cycle, we conducted an experiment consisting in the incorporation of BrdU into the newly synthesized DNA chains during the replication.

After cell fixation and preparation of metaphase spreads, the incorporated BrdU was detected via immunocytochemical staining with anti-BrdU antibodies.

**Fig. 1:**
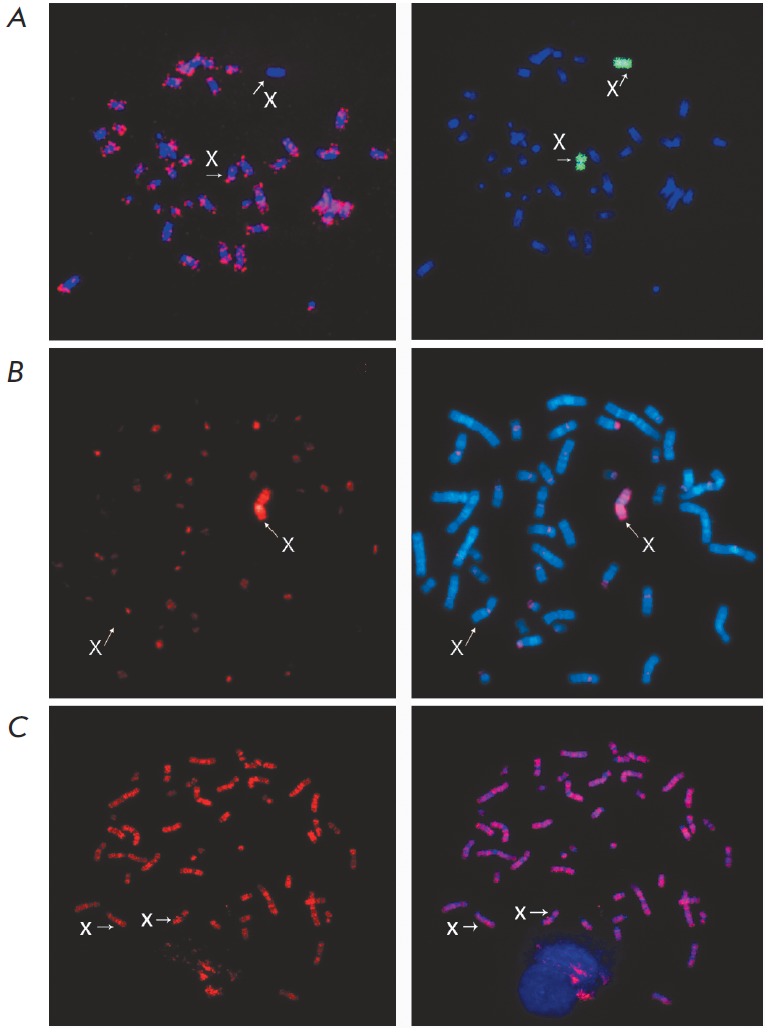
Replication pattern of X chromosomes in human pluripotent stem cells. X chromosomes are indicated by arrows and letters. A, B – Representative images of asynchronous replication of the X chromosomes. Latereplicating Xi in metaphase spread of hESM04 is shown. А – BrdU (red, left image) is incorporated in all but one chromosome. The right-hand side image represents the same metaphase after FISH with whole X chromosome probe (green). Chromosomes were stained with DAPI (blue). B – BrdU (red, left image) is incorporated only in pericentromeric constitutive heterochromatin and p- and q-arms of a single chromosome. The merged image (right) consists of BrdU (red) and DAPI (blue). X chromosomes were identified by inverted DAPI-banding (not shown). С – Representative images of the synchronous replication of the X chromosomes in the metaphase spreads of HUES9. BrdU (red, left image) is incorporated in all chromosomes but not in pericentromeric heterochromatin. The merged image (right) consists of BrdU (red) and DAPI (blue). X chromosomes were identified by inverted DAPIbanding (not shown)

The patterns of late replication of the X chromosome in all cell lines with the Xi (in the somatic HUVEC cells, in ESC (hESM01 and hESM04) and iPSC (iPS-6, iPS-7, iPS-12) lines) were obtained. *Figure 1 *shows two types of incorporation of BrdU into DNA which are observed during the late replication of Xi. Incorporation of BrdU in all chromosomes but one of the X chromosomes is observed in the first variant (*Fig. 1A*). In the second variant, BrdU is incorporated into the pericentromeric heterochromatin and in the p- and q-arms of one of the chromosomes in the metaphase plate – one of the X chromosomes, according to FISH or by inverted DAPIbanding (*Fig. 1B*). The simultaneous replication with the pericentromeric constitutive heterochromatin supports the fact that the observed incorporation type of BrdU corresponds to replication in the late S-phase.

The homologous X chromosomes in PSC lines with two Xa (HUE S 9, MA-02) replicate almost synchronously; hence, the homologous X chromosomes are almost indiscernible from one another in terms of the type of BrdU incorporation. *Figure 1C *shows an example of synchronous replication of the X chromosomes.

Next, we decided to estimate the correlation between the replication timing of the X chromosomes and the degree of compactness of their chromosome territories.

**The degree of compactness of the X chromosomes in the interphase nucleus does not necessarily correlate with the X chromosome status in human pluripotent stem cells and the timing of their replication**

**Fig. 2:**
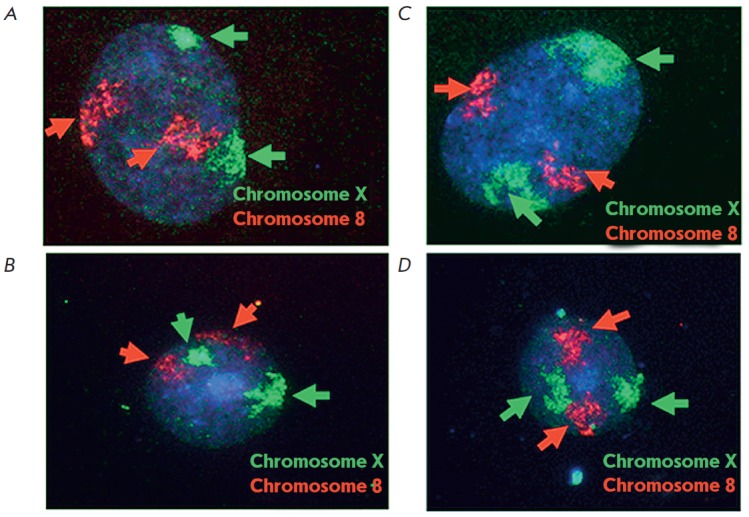
X chromosome territories in human pluripotent stem cells. A, B – The nucleus of hESM04 has one dispersed and one compact X chromosome territory; С, D – The nucleus of iPS MA02 has two dispersed X chromosome territories. Chromosomes X are green; chromosomes 8 are red; DNA is blue (DAPI); green arrows indicate the X chromosomes, red arrows indicate chromosomes 8

**Fig. 3:**
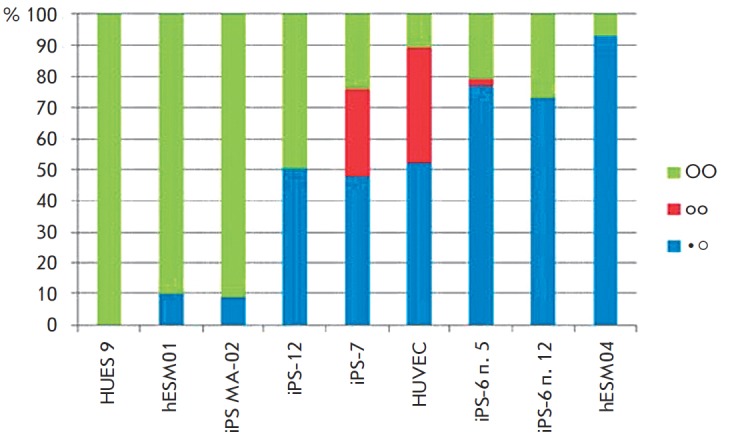
Frequencies of nuclei with different organizations of X chromosome territories in human pluripotent stem cells and in HUVEC. ●О – nuclei with one dispersed and one compact X chromosome territory (blue); ОО – nuclei with two dispersed X chromosome territories (green); оо – nuclei with two small compact X chromosome territories (“somatic” type) (red)

In order to determine the degrees of chromatin condensation, the territories of two X chromosomes of the same nucleus on flat specimens of interphase nuclei were compared. This method of specimen preparation has been used previously when studying chromosome territories [19]. A pair of autosomal chromosomes (chromosome 8) was used as a control. In mammalian cells, the autosomes have the same epigenetic status and the size of their territories is identical. The chromosome territories were compared using the algorithm (see the Materials and Methods section) and by comparing the variance values for each individual chromosome. The degrees of compactness of the X chromosome territories inside one nucleus were compared using the nuclei on which two non-overlapping zones of DNA probe hybridization to the X chromosome were well-pronounced. The nuclei of pluripotent cells were subdivided into two types without any intermediate forms: (1) nuclei with one dispersed X chromosome territory with a low staining density and one compact territory with a high staining density (*Figs. 2A,B*); (2) nuclei with two dispersed X chromosome territories with the same staining density (*Figs. 2C,D*). The results of the analysis of the distribution of pluripotent cell nuclei on the degree of compactness of the X chromosomes are shown in *Fig. 3*. The frequencies (%) of different types of nuclear organization in all cell lines are shown. The chromosome territories in pluripotent cells with two Xa were dispersed in the overwhelming majority of the analyzed nuclei (over 90%).

In cell lines where the reprogramming process was not complete (i.e., in clones not truly pluripotent: iPS-6 and iPS-7), the third type of chromatin condensation status was also observed. In this case, two X chromosome territories insignificantly differ in size and are characterized by a high degree of chromatin condensation, similarly to the autosomes in these cells and in most HUVEC cells, which originally were an object of reprogramming (*Fig. 3*). It should be mentioned that no differences in the volumes of the territories of the Xa and Xi have been detected in some studies devoted to the investigation of the arrangement of X chromosome territories in somatic cells [2, 20, 21]. The difference between the somatic and pluripotent cells can be attributed to the fact that nuclear chromatin in pluripotent cells is characterized by a considerably higher plasticity as compared with the more compact chromatin in somatic cells [1, 19]. It is interesting to mention that as the iPSC clone iPS-6 was being cultured (passages 5 through 12), the cells lost the chromosome territory of “somatic” type and most cells had one compact and one dispersed X chromosome territory in the later passage.

The results of the comparison of the degree of compactness of the X chromosomes and their replication timing are listed in *Table 2*.

Most nuclei in the hESM04 cell line had one compact and one dispersed X chromosome territory (*Fig. 2*), which was accompanied by a well-pronounced asynchronous replication of the X chromosomes. Nevertheless, no correlation between the late replication of Xi and compactness of the Xi chromosome territory has been observed in the other pluripotent cell lines.

**Table 2:** Comparison of chromosome territory folding and replication patterns of Xs

Cell lines	●О, %*	oo, %*	ОО, %*	Replication pattern
HUES9	0		100	Synchronous, early
hESM04	93		7	Asynchronous, late replication of Xi
hESM01	10		90	Asynchronous, late replication of Xi
HUVECC	52	37	11	Asynchronous, late replication of Xi
IPS-7	48	28	24	Asynchronous, late replication of Xi
IPS-6 p.5	77	2	21	Asynchronous, late replication of Xi
IPS-6 p.12	73		27	Asynchronous, late replication of Xi
IPS-12	50		50	Asynchronous, late replication of Xi
iPS MA02	3		97	Synchronous, early

* ●О – nuclei with one dispersed and one compact X chromosome territory; оо – nuclei with two small compact X chromosome territories (“somatic” type); ОО – nuclei with two dispersed X chromosome territories.

As can be seen in *Table 2*, despite the fact that both X chromosome territories in the ESC hESM01 line were dispersed, replication of the X chromosomes in this cell line occurred asynchronously, with one of the X chromosomes replicating in the late S-phase. In the iPSC clone iPS-12, half of the nuclei had two identical, dispersed X chromosome territories; nevertheless, despite the partial cytological signs of reactivation (the pres ence of H3K4me2, *Table 1*), one of the X chromosomes replicated in the late S-phase in all cells analyzed (*N *> 20) and possessed the Xi status.

The ESC HUE S 9 and iPSC MA-02 lines, in which the homologous X chromosomes replicated almost synchronously and neither of the X chromosome was characterized by late replication, had two dispersed X chromosome territories (in all nuclei for HUE S 9 and in most nuclei for MA-02) (*Table 2*).

Thus, summarizing the data obtained for all the cell lines used in this study, one can assume that the Xi sta tus can be retained in pluripotent cells without the formation of the conventional “Barr body” (i.e., the compactly packed chromosome territory). Nevertheless, the active status of both X chromosomes and the transition to synchronous replication during re-programming require a dispersed status of both X chromosomes in the interphase nucleus.

During the reprogramming to a pluripotent state in the cases when partial or complete Xi reactivation occurs, dispersion of the chromosome territory is likely to take place before the major heterochromatin marks H3K27me3 and H3K9me3 disappear (as it can be seen by the example of the iPSC clone iPS-12).

It has been demonstrated that long-term cultivation of partially reprogrammed iPSCs frequently promotes the completion of reprogramming, the acquisition of a pluripotent state, and a loss of characteristics by somatic cells. The change in the compactness of X chromosome territories, which occurred during the cultivation of the iPSC clone iPS-6, demonstrates that this parameter can be an additional marker of the reprogramming of somatic cells to a pluripotent state.

## CONCLUSIONS

It has been demonstrated in our studies that human PSCs with Xi can possess either dispersed or compact chromosome territory of Xi. ESC lines with both X chromosomes active or iPSC lines in which the X chromosome has been reactivated during reprogramming have dispersed both of the X chromosome territories in the interphase nucleus.

Thus, a conclusion can be drawn that human PSCs have a mechanism that allows them to maintain an inactivated status of the X chromosome, which does not depend directly on the degree of compactness of its chromosome territory in the interphase nucleus. Late replication of the inactivated X chromosome is also independent of its degree of compactness.

On the other hand, the X chromosome territory has to be dispersed for the X chromosome to become active during its reactivation and synchronous replication.

## Abbreviations

BrdU: 5-bromo-2’-deoxyuridine
DAPI: 4’, 6-diamidino-2-phenylindole
ESCs: embryonic stem cells
FISH: fluorescent in situ hybridization
H3K27me3: trimethylation of histone H3 at lysine 27
H3K4me2: dimethylation of histone H3 at lysine 4
H3K9me3: trimethylation of histone H3 at lysine 9
HUVEC: human umbilical vein endothelial cells
iPS cells: induced pluripotent stem cells
Xa: active X chromosome
Xi: inactive X chromosome
PSC: pluripotent stem cells
